# The Roles and Interactions of Symbiont, Host and Environment in Defining Coral Fitness

**DOI:** 10.1371/journal.pone.0006364

**Published:** 2009-07-24

**Authors:** Jos C. Mieog, Jeanine L. Olsen, Ray Berkelmans, Silvia A. Bleuler-Martinez, Bette L. Willis, Madeleine J. H. van Oppen

**Affiliations:** 1 Australian Institute of Marine Science, Townsville, Queensland, Australia; 2 Department of Marine Benthic Ecology and Evolution, Centre for Ecological and Evolutionary Studies, Biological Centre, University of Groningen, Haren, The Netherlands; 3 Institute of Microbiology, Eidgenössische Technische Hochschule Zürich (ETH), Zürich, Switzerland; 4 ARC Centre of Excellence for Coral Reef Studies and School of Marine and Tropical Biology, James Cook University, Townsville, Queensland, Australia; University of North Carolina at Chapel Hill, United States of America

## Abstract

**Background:**

Reef-building corals live in symbiosis with a diverse range of dinoflagellate algae (genus *Symbiodinium*) that differentially influence the fitness of the coral holobiont. The comparative role of symbiont type in holobiont fitness in relation to host genotype or the environment, however, is largely unknown. We addressed this knowledge gap by manipulating host-symbiont combinations and comparing growth, survival and thermal tolerance among the resultant holobionts in different environments.

**Methodology/Principal Findings:**

Offspring of the coral, *Acropora millepora*, from two thermally contrasting locations, were experimentally infected with one of six *Symbiodinium* types, which spanned three phylogenetic clades (A, C and D), and then outplanted to the two parental field locations (central and southern inshore Great Barrier Reef, Australia). Growth and survival of juvenile corals were monitored for 31–35 weeks, after which their thermo-tolerance was experimentally assessed. Our results showed that: (1) *Symbiodinium* type was the most important predictor of holobiont fitness, as measured by growth, survival, and thermo-tolerance; (2) growth and survival, but not heat-tolerance, were also affected by local environmental conditions; and (3) host population had little to no effect on holobiont fitness. Furthermore, coral-algal associations were established with symbiont types belonging to clades A, C and D, but three out of four symbiont types belonging to clade C failed to establish a symbiosis. Associations with clade A had the lowest fitness and were unstable in the field. Lastly, *Symbiodinium* types C1 and D were found to be relatively thermo-tolerant, with type D conferring the highest tolerance in *A. millepora*.

**Conclusions/Significance:**

These results highlight the complex interactions that occur between the coral host, the algal symbiont, and the environment to shape the fitness of the coral holobiont. An improved understanding of the factors affecting coral holobiont fitness will assist in predicting the responses of corals to global climate change.

## Introduction

The obligate symbiosis between reef-building corals and unicellular algae of the genus *Symbiodinium*, commonly referred to as zooxanthellae, is a key feature of tropical coral reefs. The algal endosymbionts are photosynthetically active, and provide up to 95% of the energy requirement of the coral host [1]. In return, the coral host offers protection from predation and an environment with increased inorganic nutrients [2]. The success of coral reefs and their capacity to thrive in oligotrophic tropical waters has been heavily dependent on this partnership. The coral-zooxanthellae symbiosis is very sensitive to increases in temperature, however, and changes of as little as 1°C above the average summer maximum can lead to a breakdown of the symbiosis. This breakdown results in expulsion and/or degradation of the algal partner causing the phenomenon known as coral bleaching (reviewed by Coles and Brown [3]). When bleaching is severe, and the symbiosis is unable to re-establish itself, the coral dies.

The genus *Symbiodinium* is highly diverse and consists of eight phylogenetic clades with each containing multiple subclades/types [4]–[6]. Scleractinian corals form symbioses with members of six of these clades (A–D, F, G), but predominantly with those of clades A–D [7], [8]. This genetic diversity is reflected functionally in traits that vary with symbiont type, such as growth and thermal tolerance of the holobiont, as well as the photosynthetic response of both *in* and *ex hospite* zooxanthellae [9]–[16]. Although several previous studies have experimentally controlled for host and environmental factors, no study to date has compared the performance of coral symbioses with varying symbiont and host genotypes under different environmental conditions in the field, nor the extent to which holobiont traits are affected by either the host or symbiont [11], [17], [18]. A better understanding of genotype x environment interactions is essential for predicting the potential of the holobiont to acclimatize to global warming through changes in the algal symbiont community [19]–[21] and adaptation through selection on coral holobiont traits [22].

Most corals produce zooxanthella-free larvae, with each generation acquiring algal symbionts anew from the environment [23]. Multiple *Symbiodinium* types are typically taken up by juvenile corals [13], [24], with mostly one type becoming dominant over time [8], [25]. The other types are often not lost completely, but are reduced to low abundances or background densities that can persist throughout adult life [26]. Changes in the *Symbiodinium* population of adult corals may be realized, therefore, through an increase in the relative abundance of these background types. For example, sub-lethal bleaching events can result in changes in the proportion of different algal types leading to dominance of the association by more thermo-tolerant *Symbiodinium* types [27]. Alternatively, adult corals may take up exogenous symbionts from the water column to establish a new symbiosis. This process has been documented under experimental conditions for anemones [10] and octocorals [28], but is expected to be more restricted in scleractinian corals [29]. Symbiont change within a coral population can theoretically also stem from uptake of a new symbiont type from one generation to the next [30], but this has not been documented experimentally.

Here, we present results from a reciprocal grow-out experiment involving two populations of the common scleractinian coral *Acropora millepora* from two thermally contrasting, inshore environments on the Great Barrier Reef (Magnetic Island and the Keppel Islands). Individuals from each location were allowed to spawn in the laboratory to produce azooxanthellate juveniles, which were subsequently exposed to six different *Symbiodinium* types from three phylogenetic clades (A/C2* mixture, C1, C2, C• and D). The new holobionts were then returned to the field and fitness parameters measured over 31+weeks. The data show that the holobiont fitness traits growth, survival and thermal tolerance are differentially affected by the source population of the coral host, symbiont type and environmental factors, and that trade-offs between these fitness traits vary with environmental conditions.

## Results

### Establishment and stability of symbioses with the different algal partners

Newly settled, azooxanthellate polyps of *A. millepora*, which had been offered six different symbiont types, successfully established symbioses with *Symbiodinium* types C1, D and C2*/A in juvenile cohorts originating from both the Magnetic Island and Keppel Islands populations. This was indicated by large numbers of *Symbiodinium* cells in coral juvenile squash preparations. In contrast, no zooxanthellae were found in squash preps of either the C2 or C• treatments, indicating that no symbioses were established. SSCP analyses of ethanol preserved squash preparations showed that juveniles exposed to a mixture of *Symbiodinium* C2* and A established symbioses with *Symbiodinium* A symbionts only.

The nomenclature of experimental groups consisted of a three-letter code designating the location of the outplant, the location of the parental population, and the *Symbiodinium* type. Genetic analyses of juveniles outplanted to Magnetic Island and the Keppel Islands at several time points revealed that symbioses with *Symbiodinium* C1 and D were stable over the 31+weeks of this study at both locations (supporting information, Table S3). In contrast, after 9–13 weeks, only *Symbiodinium* D was found in: MMA juveniles (Magnetic Island juveniles inoculated with *Symbiodinium* A and outplanted to Magnetic Island), MKA juveniles (Keppel Island juveniles inoculated with *Symbiodinium* A and outplanted to Magnetic Island), and the uninfected groups (those exposed to C2 or C•) at both locations (apart from a single colony in the latter group at the Keppel Islands containing both C1 and D). KKA juveniles (Keppel Island juveniles inoculated with *Symbiodinium* A and outplanted to Keppel Islands) continued to harbor mostly *Symbiodinium* A for 31 weeks, but 30% of the colonies were found to harbor mixtures of A and C1 and/or D at the end of this period.

### Growth and survival of outplanted juvenile corals

**Figure 1:**
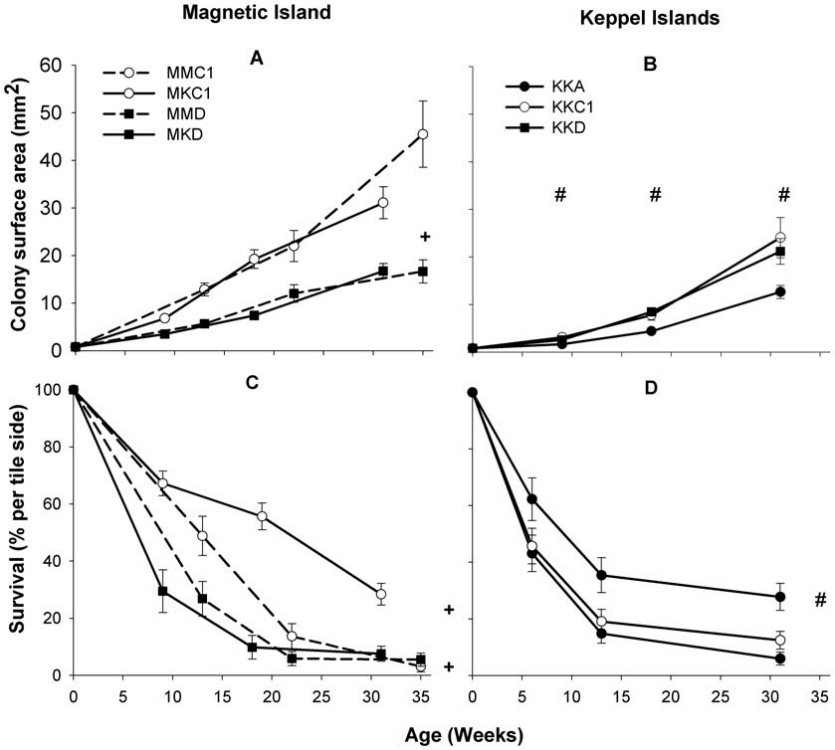
Growth and survival of coral juveniles at Magnetic Island (a+c) and the Keppel Islands (b+d). See materials and methods for nomenclature of the experimental groups. +indicates significant difference between juvenile corals harboring *Symbiodinium* C1 and those harboring D (p<0.05), and # indicates significant difference between KKA and KKC1/KKD corals (growth, p<0.05) or between KKD and KKA/KKC1 corals (survival, p<0.001). C1 corals grew and survived better at Magnetic Island than the D corals. At the Keppel Islands, KKC1 and KKD corals grew at similar rates, but KKD corals had a better survival rate. KKA corals grew slowest at the Keppel Islands and had a low survival rate.

Patterns in growth rates of *A. millepora* juveniles associated with C1 or D symbionts differed significantly between Magnetic Island and the Keppel Islands (p<0.05, Table 1a), indicating that the effect of symbiont type on coral growth differed between the two outplant locations (Fig. 1a, b). At Magnetic Island, the C1 corals (MMC1 and MKC1) grew nearly twice as fast as the D corals (MMD and MKD) (Fig. 1a, p<0.05), whereas at the Keppel Islands no difference in growth rate was found between KKC1 and KKD corals (Fig. 1b). KKA corals, however, grew significantly slower than either KKC1 or KKD corals (Fig. 1b and Table 1b, p<0.001).

**Table 1:** Results of ANOVA analyses. GLM = General Linear Model, RM = Repeated Measures, F = Factorial.

Predictor(s)	Type 3 SS	df	Se	f	p
**a) GLM-ANOVA: effect of symbiont type, host population and outplant location on growth**					
Symbiont	0.393	1	0.393	6.195	0.015*
Host pop	0.002	1	0.002	0.036	0.850
Outpl. loc	0.041	1	0.041	0.651	0.422
Symbiont*Outpl. loc	0.350	1	0.350	5.521	.021*
Symbiont*Host pop	0.050	1	0.050	0.783	0.379
**b) RM-ANOVA: effect of symbiont type on growth over time at the Keppel Islands**					
Time	14.84	2	7.418	307.20	0.000*
Symbiont	1.47	2	0.736	11.00	0.000*
Time*Symbiont	0.11	4	0.026	1.10	0.37
**c) RM-ANOVA: effect of temperature, symbiont type and host population on PSII excitation pressure over time (heat-stress experiment 1)**					
Time	0.272	10	0.027	31.280	0.000*
Time*Temp	0.496	30	0.017	18.980	0.000*
Time*Symbiont	0.099	10	0.010	11.410	0.000*
Time*Host pop	0.016	10	0.002	1.790	0.061
Time*Temp*Symbiont	0.234	30	0.008	8.970	0.000*
Time*Temp*Host pop	0.024	30	0.001	0.940	0.567
**d) F-ANOVA: effect of temperature, symbiont type and host population on relative symbiont densities (heat-stress experiment 1)**					
Temp	615.100	3	205.000	41.890	0.000*
Symbiont	18.500	1	18.500	3.780	0.056
Host pop	6.900	1	6.900	1.410	0.239
Temp*Symbiont	8.200	3	2.700	0.560	0.646
Temp*Host pop	10.400	3	3.500	.07410	0.549
Temp*Host pop*Symbiont	23.800	3	7.900	1.620	0.191
**e) RM-ANOVA: effect of temperature and outplant location on PSII excitation pressure over time (heat-stress experiment 2)**					
Time	0.050	7	0.007	21.200	0.000*
Time*Temp	0.020	14	0.001	4.300	0.000*
Time*Outpl. loc	0.004	7	0.001	1.800	0.091
Time*Temp*Outpl. loc	0.005	14	0.000	1.100	0.342
**f) RM-ANOVA: influence of temperature and symbiont type on PSII excitation pressure over time (heat-stress experiment 2)**					
Time	1.131	7	0.162	32.420	0.000*
Time*Temp	1.172	14	0.084	16.800	0.000*
Time*Symbiont	0.964	14	0.069	13.810	0.000*
Time*Temp*Symbiont	1.510	28	0.054	10.820	0.000*
**g) F-ANOVA: influences of temperature and outplant location on rel. symbiont densities (heat-stress experiment 2)**					
Temp	284.300	3	94.770	12.820	0.000*
Outpl. loc	3.300	1	3.290	0.450	0.508
Temp*Outpl. loc	32.000	3	10.660	1.440	0.245
**h) F-ANOVA: influences of temperature and symbiont type on rel. symbiont densities (heat-stress experiment 2)**					
Temp	222.800	3	74.300	8.770	0.000*
Symbiont	209.500	2	104.800	12.360	0.000*
Temp*Symbiont	271.300	6	45.200	5.340	0.000*

Symbiont type also had a significant effect on survival, for example C1 corals survived better than D corals at Magnetic Island (Fig. 1c, MMC1 * MMD: p<0.05, MKC1 * MKD: p<0.001). This was especially evident in the first 12 weeks. As the coral juveniles matured, host-correlated differences became evident between MMC1 and MKC1 corals, with the latter corals having better survival than MMC1 corals (not statistically tested because of age difference). At the Keppel Islands, the pattern was opposite to that at Magnetic Island, with survival being significantly higher for KKD than for either KKA or KKC1 (Fig. 1d, p<0.001). Hence, survival was also affected by the outplant location (Fig. 1c,d).

### Laboratory heat-stress experiments

#### Experiment 1

**Figure 2:**
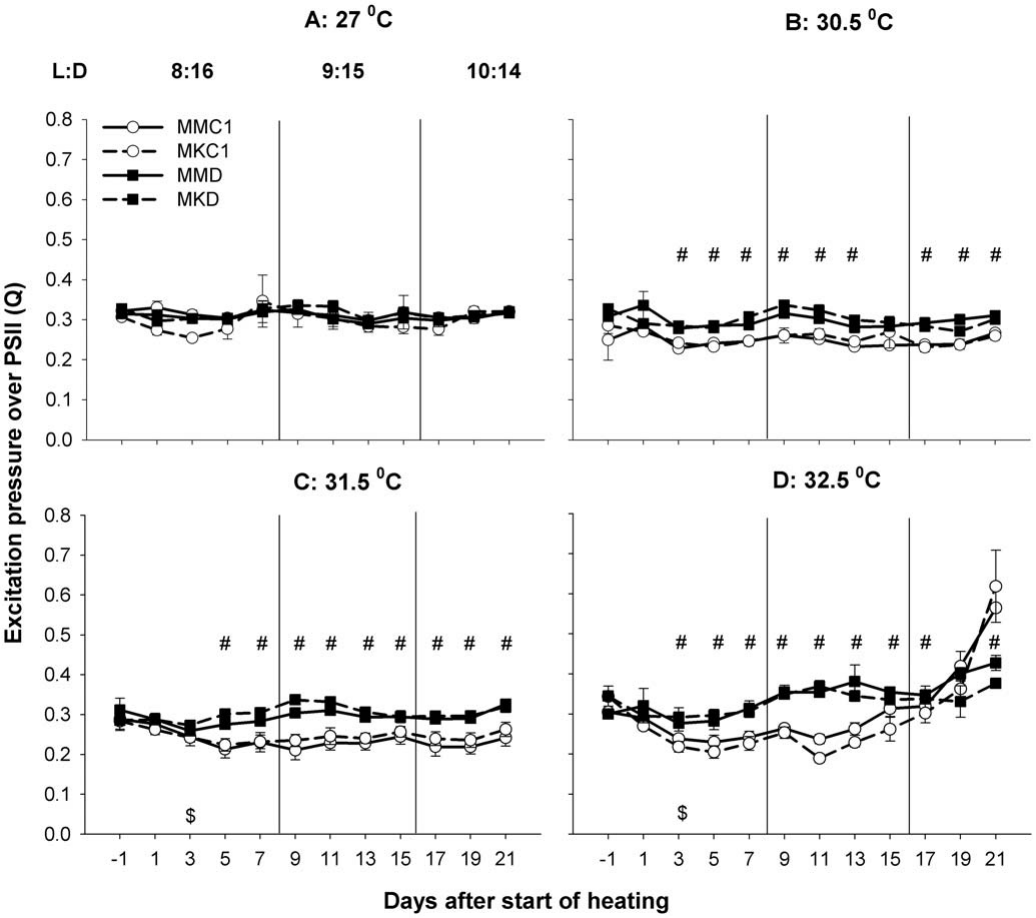
Heat-stress experiment 1: PAM-fluorometry. Effect of four different temperature regimes on the excitation pressure over photosystem II of four groups of juvenile coral outplanted to Magnetic Island. See materials and methods for nomenclature. L:D = light-dark regime, $ = target temperature is reached, # = significant difference between C1 corals and D corals. C1 corals responded stronger to the highest temperature treatment than D corals, as indicated by a stronger increase in Q for C1 corals towards the end of the experiment.

This experiment compared the thermal tolerances of four coral groups outplanted to Magnetic Island (MMC1, MMD, MKC1, MKD corals). There was a significant difference in photosynthetic performance, measured as the excitation pressure on PSII (Q), between C1 corals (MMC1 and MKC1 corals) and D corals (MMD and MKD corals, Fig. 2 and Table 1c). In contrast, no significant effect of host population origin (i.e., host genetic background) over time was found (Table 1c). At the intermediate temperatures (30.5 and 31.5°C, Fig. 2b, c), the Q of C1 corals decreased at the beginning of the experiment, whereas the Q of D corals remained mostly level, resulting in a significantly lower Q for C1 corals (p<0.05) for most of the experiment. Exposure to 32.5°C (Fig. 2d) initially resulted in a similar reduction of Q in C1 corals (not seen in the D corals), but after ∼11 days of exposure, Q increased in the C1 corals to exceed the Q of D corals by the end of the experiment (p<0.05). The Q of the D corals showed a smaller increase at the end of the experiment. These results were interpreted to indicate a lower thermo-tolerance of C1 corals compared to D corals. This difference in thermo-tolerance was further supported by an earlier and stronger reduction in Fv/Fm for C1 corals than for D corals at 32.5°C (supporting information, Fig. S3).

**Figure 3:**
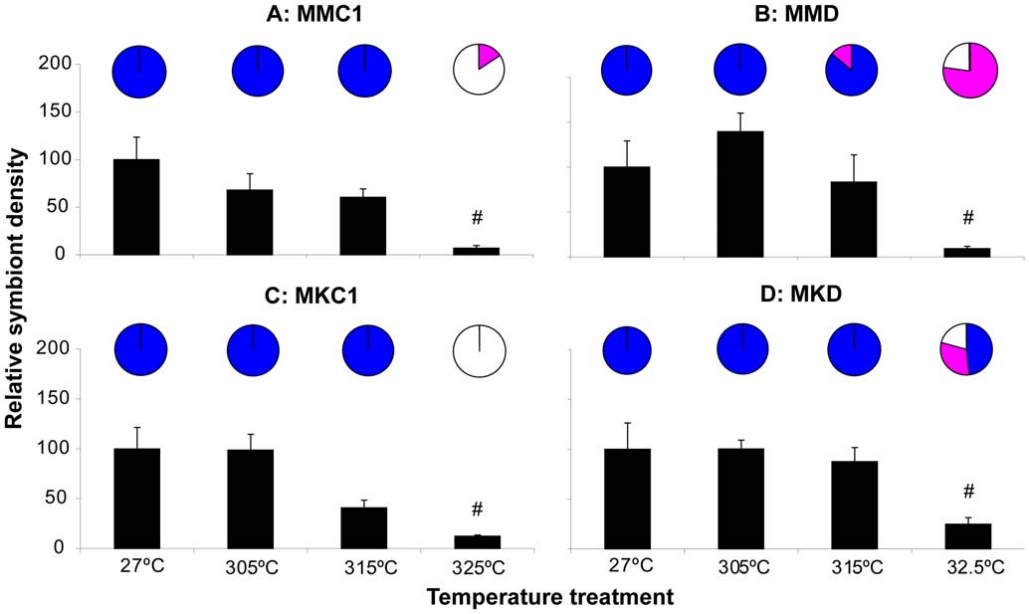
Heat-stress experiment 1: relative algal symbiont densities and coral condition. End effect of four different temperature regimes on the relative algal symbiont densities (bars) and coral condition (pies) of four groups of juvenile coral outplanted to Magnetic Island. See materials and methods for nomenclature. Blue = healthy, purple = pale, white = bleached. # = significantly different from lower temperatures within a group (p<0.05). All four experimental groups exhibited a bleaching response at the highest temperature treatment, as indicated by significant reductions in relative algal symbiont densities, and the visual assessment indicated a stronger response (more bleached colonies) for C1 corals than for D corals.

*Symbiodinium* cell density measurements showed significant temperature-related reductions by the end of the experiment in all groups (Fig. 3a–d bar graphs, Table 1d, p<0.0001), indicating that all groups experienced significant bleaching at the highest temperature. No Temperature*Symbiont Type effect was found for *Symbiodinium* density (Table 1d), but visual assessment of coral color suggested a stronger bleaching response at 32.5°C in C1 corals than in D corals (more C1 colonies had a bleached appearance, Fig. 3a–d pie graphs).

#### Experiment 2

**Figure 4:**
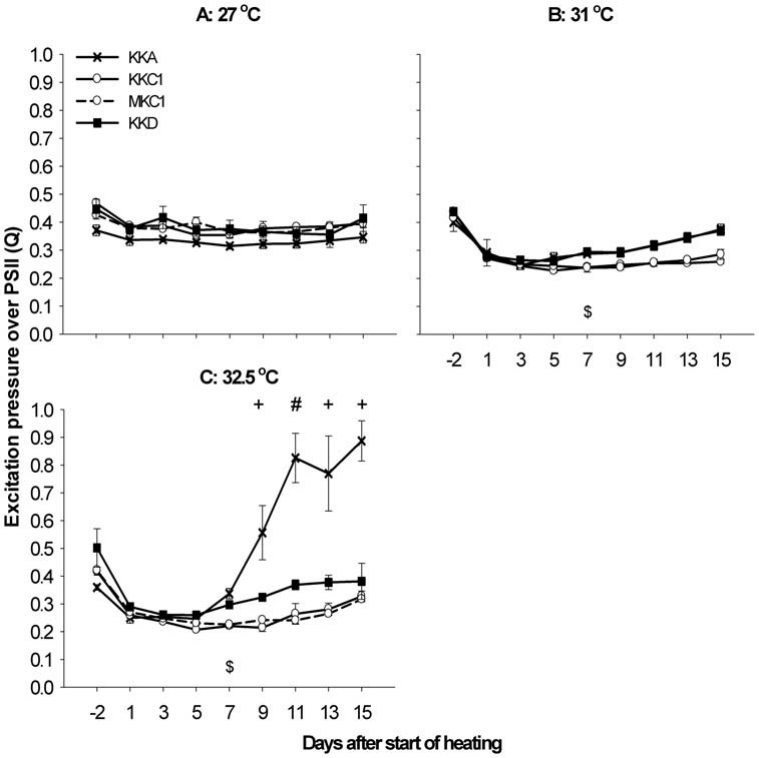
Heat-stress experiment 2: PAM-fluorometry. Effect of three different temperature regimes on the excitation pressure over photosystem II of three groups of juvenile coral outplanted to the Keppel Islands, and one outplanted to Magnetic Island. See materials and methods for nomenclature. $ = target temperature is reached, + = significant difference between KKA and MKC1/KKC1/KKD corals (p<0.001), # = significant difference between all three symbiont types (p<0.05). KKA corals responded much stronger to the heat-stress than the other three juvenile coral groups, as indicated by a sharp increase in Q values for KKA corals in the highest temperature treatment relatively early in the experiment.

This experiment assessed the thermal tolerance of three coral groups outplanted to the Keppel Islands (KKA, KKC1, KKD corals) and one group outplanted to Magnetic Island (MKC1 corals). There was a strong symbiont type effect on Q under heat-stress (Fig. 4a–c and Table 1f, p<0.001). In contrast, no significant effect of outplant location was found; the KKC1 and MKC1 corals responded in a similar manner at all temperatures and time points (Table 1e). No significant differences in Q were found between the experimental groups at 27°C or 31°C (Fig. 4a, b), although at 31°C a different trend was visible for KKC1/MKC1 vs KKA and KKD. All groups responded immediately to the heating by a strong reduction in Q. Next, KKC1/MKC1 remained level for the duration of the experiment, whereas KKA and KKD showed a slow but steady increase over the next two weeks. At the highest temperature (32.5°C, Fig. 4c), all groups initially responded again with a strong reduction in Q. Next, Q rapidly increased for KKA corals after ∼1 week and approached values of 1 by the end of the experiment. This coincided with a sharp drop in maximum quantum yield (supporting information, Fig. S4), indicating severe heat-stress in the KKA group early in the experiment. Variance around the mean in Q for KKA after 9 days was relatively high, due to nine colonies within the KKA group that were less heat-stressed. These colonies were sampled at the end of the experiment, and upon genotyping, were found to harbor a residual community of type D *Symbiodinium*. By comparison, Q values of KKC1/MKC1 and KKD corals were much less affected by the cumulative heat-stress: KKC1/MKC1 showed a small increase at the end of the experiment, whereas KKD showed an earlier small increase and leveled out from day 11 onwards. The maximum quantum yield showed relatively small and similar reductions for KKC1/MKC1 and KKD corals (supporting information, Fig. S4).

**Figure 5:**
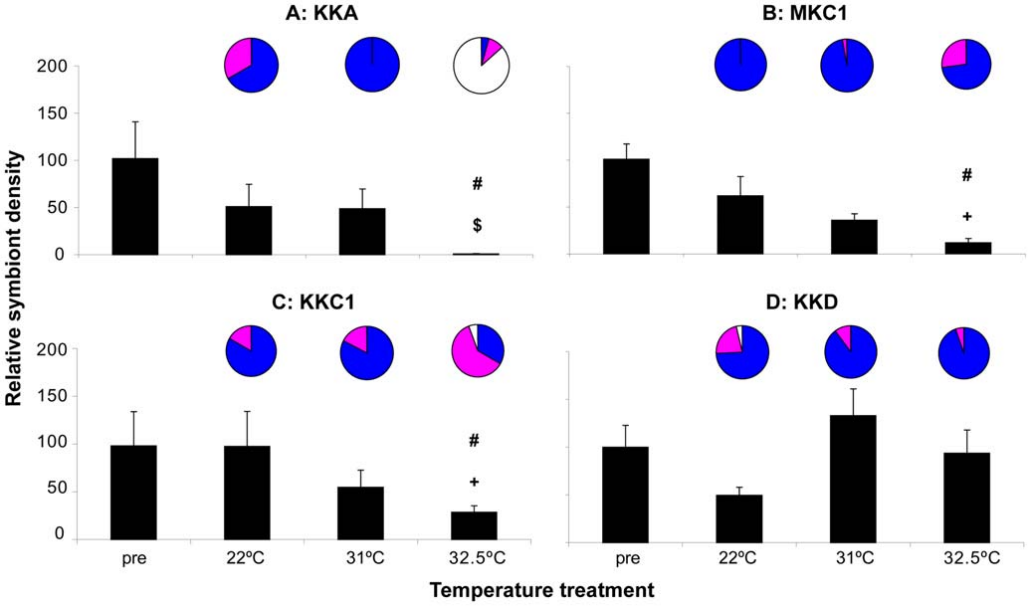
Heat-stress experiment 2: relative algal symbiont densities and coral condition. End effect of three different temperature regimes on the relative algal symbiont densities (bars) and coral condition (pies) of three groups of juvenile coral outplanted to Magnetic Island, and one outplanted to Magnetic Island. Blue = healthy, purple = pale, white = bleached. # = significantly different from lower temperatures within the same group (p<0.05), $ = significantly different from KKC1/MKC1 and KKD corals at the same temperature (p<0.05), + = significantly different from KKA and KKD corals at same temperature (p<0.05). KKA corals showed the strongest bleaching response as indicated by the strongest reduction in relative algal symbiont densities and bleached appearances, KKC1/MKC1 corals showed an intermediate bleaching response as indicated by intermediate reductions in relative algal densities, and KKD corals showed no signs of a bleaching response.

*Symbiodinium* cell densities did not differ significantly between C1 corals originating from the two host populations (KKC1 vs MKC1 corals) across the different temperatures (Fig. 5a–d, Table 1g). However, there was a significant Temperature*Symbiont Type interaction with corals associated with *Symbiodinium* type A being more affected than those with C1, which in turn were more affected than those with D at the highest temperature (Table 1h). At 32.5°C, almost no *Symbiodinium* type A could be detected at the end of the experiment (Fig. 5a) while symbiont densities were also significantly reduced in the C1 corals (MKC1 and KKC1 corals; Fig. 5b, c). In contrast, symbiont densities were only marginally lower in the KKD corals (Fig. 5d). This was in agreement with the visual appearances of the holobionts: KKA corals were almost all bleached, KKC1 had a few bleached colonies (not seen for MKC1), and most KKD corals appeared healthy.

## Discussion

### Factors affecting coral holobiont fitness

This study shows that, for the scleractinian coral *Acropora millepora*, *Symbiodinium* identity is the strongest predictor of coral holobiont fitness as assessed by growth, survival and thermal tolerance. Growth and survival were secondarily shaped by environmental conditions experienced during early development from a single-polyp to a multi-polyp stage at the outplant locations. In contrast, almost no host population (i.e., host genetic) effects were evident in the three traits measured (growth, survival, heat-tolerance), even though the populations are genetically distinct based on analysis of variation at a set of allozyme loci [31]. Smith *et al.* [32] found a similar lack of host genetic influence on skeletal growth of *Pocillopora eydouxi* in a reciprocal transplant experiment. Note that in interspecific comparisons, host factors are expected to play an important role in shaping the differential fitness of coral holobionts [33], [34].

Acclimatization is often reversible, but in some cases it may become fixed early in ontogeny, which is referred to as developmental plasticity or irreversible non-genetic adaptation [35]. Developmental plasticity in thermo-tolerance has been found in organisms such as *Drosophila* sp. [36] and zebrafish [37], but our study is the first to assess developmental plasticity in a coral species, which has the added complexity of being a symbiotic association. We could only assess developmental plasticity in thermal tolerance after laboratory acclimation, as the two other traits were measured in the field. The almost identical response to thermal stress in holobionts grown out in two different environments suggests an absence of developmental plasticity for thermo-tolerance.

Environmental factors associated with the two outplant sites determined whether trade-offs due to associating with different symbiont types were realized. The trade-off found at Magnetic Island between thermo-tolerance and growth/survival when juveniles were associated with *Symbiodinium* C1 versus D (see also [13]), was absent in the Keppel Islands. This variability in the realization of trade-offs has important implications for the potential of symbiont shuffling [7], [21] as a mechanism to induce lasting changes in coral holobiont physiology. After shuffling, post-stress reversals [38] may occur when the fitness of the thermo-tolerant symbiont is lower than that of the pre-stress symbiont in the absence of stress. Our results suggest that the potential for symbiont shuffling to increase holobiont fitness may be dependent on the environment. In light of a modeling study, which showed that trade-offs are important in the evolution of bleaching resistance in corals [22], understanding differences in symbiont-linked trade-offs between reef populations is important for assessments of reef resilience.

### A thermal-tolerance ranking for *A. millepora-Symbiodinium* associations

*A. millepora* juveniles associated with *Symbiodinium* A were the least thermo-tolerant of the three coral-*Symbiodinium* associations tested, based on their inability to maintain the association at Magnetic Island and experimental evidence of greatest impact of heat stress on *Symbiodinium* A corals, i.e.: the sharp increase in Q recorded for KKA corals (not seen in KKC1/MKC1 or KKD corals), their bleached appearance, and large reductions in Fv/Fm and relative symbiont densities. Although subtle, the combined results from experiments 1 and 2 indicated that C1 corals are less thermo-tolerant than the D corals in this species. In experiment 1, the larger increase in Q of the C1 corals in the 32.5°C treatment indicated a stronger stress response for this group compared to the D corals. This interpretation was supported by earlier and stronger reduction in Fv/Fm and a larger number of bleached colonies at the end of the experiment for C1 corals than for D corals. However, the relative zooxanthella densities were similarly reduced for all coral groups, indicating that all groups exhibited a bleaching response. At the lower cumulative heat-stress level of experiment 2, relative symbiont densities indicated that the C1 but not the D corals bleached to some extent, but no significant difference in thermo-tolerance between the C1 and D corals was evident from the Q or Fv/Fm measurements (although different trends were visible, see below). Both apparent inconsistencies may be explained by the fact that samples for relative symbiont density determinations were taken one day after the last PAM-measurements, leading to a stronger heat-stress effect on symbiont density than on fluorescence. Alternatively, loss of symbiont cells due to heat-stress may have preceded large responses in the fluorescent parameters. Whatever the cause, the difficulty in separating the thermo-tolerance of C1 and D indicates that the differences are small.

The consistently lower Q of C1 corals compared to D corals at relatively low levels of accumulated heat-stress during heat-stress experiment 1 resulted from a decrease in Q of C1 corals as an initial response to the temperature increases. The relative symbiont density measurements and visual assessments indicated that these (temperature-induced) differences were unrelated to bleaching. The early increase in Q at 32.5°C for D corals in experiment 2 (after the initial drop for all groups) to higher values than for C1 corals during relatively low levels of accumulated heat-stress was similarly uncorrelated with bleaching, as were the increasing trends in KKA and KKD at 31°C. The increase in Q for C1 corals (exp. 2) at higher accumulated heat-stress (last four days) however was correlated with decreased symbiont densities. Because Q takes into account both the photochemical and non-photochemical processes [39], temperature affects Q in multiple ways and the changes in this parameter may therefore not always be related to heat-stress, especially when they occur at low levels of accumulated heat-stress and remain at low values. For instance, dark reaction enzymes of photosynthesis increase the rate of catalyzed reactions with temperature (up to temperatures causing protein damage) [40], and a reduction in closed reaction centres with increasing temperature under the same irradiance could therefore be expected. Importantly, these effects may differ between symbiont types. We would therefore suggest that Q data are better interpreted as a change over time (and with accumulative heat-stress), and assessed in conjunction with other parameters, such as symbiont densities and visual assessments.

We were unable to raise holobionts with the generalist symbiont type C2 (Lajeunesse *sensu* C3), one of the main symbiont types on the GBR [41]–[43]. However, it is known that both C1 and D are more common than C2 at relatively warm, inshore locations [41], [44], C2 confers a 1–1.5°C lower thermo-tolerance in adult *A. millepora* than D [12], and both C1 and D increased in relative abundance at the expense of C2 after a natural bleaching event [27]. Taken together, this strongly suggests that C2 confers a significantly lower thermo-tolerance to *A. millepora* than either C1 or D. Its relative tolerance in comparison to A remains to be determined. Therefore, we can rank the thermo-tolerance of *A. millepora*-*Symbiodinium* associations as D>C1≫C2/A. Importantly, Abrego *et al.* [34] found that *Acropora tenuis* had a higher thermo-tolerance with *Symbiodinium* C1 rather than with D, indicating that this ranking may differ between coral species.

### *Symbiodinium* type A is a suboptimal symbiont

The *Symbiodinium* type A used here belongs to subclade A1 [45] which has been found worldwide (e.g. the Caribbean, Red Sea, French Polynesia, Bermuda, Japan, the Great Barrier Reef) in a variety of hosts including scleractinian corals, zoanthids, jellyfish and giant clams [45]–[48]. Recently, it has been suggested that members of the clade A lineage may be more adapted to a free-living life-style and have opportunistic interactions with cnidarian hosts such as corals, which may more resemble parasitism [49]. This conclusion was based on (1) the relative rarity of coral-clade A associations (e.g. [42]), (2) the presence of clade A in corals with a reduced health [49]–[51], (3) low carbon translocation to hosts when in symbiosis with clade A compared to clade C [49], (4) low diversity within clade A, suggesting an opportunistic lifestyle [49], and (5) clade A symbionts outcompete other clades in culture [52]. Our results support the notion that clade A-coral associations are correlated with poor coral health, and are of a relatively unstable, opportunistic nature. In contrast to our results, Robison & Warner [16] found that A1 (obtained from the jellyfish *Cassiopea xamachana*) was relatively thermo-tolerant based on experiments on long-running *Symbiodinium* cultures. However, no clade C or D *Symbiodinium* were included in these experiments, and responses of *Symbiodinium* in culture and *in hospite* are known to differ [11], making comparisons with our results difficult.

### Specificity in uptake of experimentally delivered symbionts

The majority of coral-*Symbiodinium* partnerships—including *A. millepora—*exhibit horizontal symbiont transmission. An advantage of this mode of transmission is that coral juveniles are able to form partnerships that are best adapted to the local environmental conditions [20], [30]. Several studies have shown that the initial acquisition of symbionts by cnidarian juvenile hosts is relatively non-specific. Symbiont specificity develops later in the development of the host (reviewed by Thornhill *et al.* [53]). Certain *Symbiodinium* types from clades A, C and D are similarly successful in infecting juveniles of *A. millepora* (this study) and *A. longicyathus* [54]. In contrast, inoculation with *Symbiodinium* C2, C2* and C• did not result in infection of the *A. millepora* juveniles in our experiments. The inability of C• (C15 *sensu* Lajeunesse *et al.* [43]) to establish a symbiosis was not unexpected, as this type has mostly been found in the maternally transmitting coral genera *Montipora* and *Porites* [43], [55]–[57] and direct symbiont transfer from generation to generation favors the evolution of specialist symbiont lineages [58]. However, the failure of the C2/C2* types to infect the coral juveniles was unexpected, since these are among the most common types found in (adult) *A. millepora* populations on the GBR [41]. We have no explanation for these results, and can only hypothesize that (1) the physical conditions of our experimental setup were unfavorable for C2 and C2*, and/or (2) *Symbiodinium* C2 and C2* are taken up at a later developmental stage in nature. Interestingly, the ‘uninfected’ juveniles that were outplanted to the Keppel Islands mostly took up *Symbiodinium* D in the first few months and no *Symbiodinium* C2 was found in any of the genotyped samples, supporting the hypothesis that developmental stage might play a role in establishment of the C2/C2*-symbioses.

### Conclusions and future directions

This study reveals that the fitness of *A. millepora* in GBR populations is primarily influenced by the symbiont type(s) it harbors, and secondarily by environmental factors. In contrast, host population origin, and hence host genetic differences, were shown to have limited effect on growth and survival. No evidence for developmental plasticity of thermo-tolerance was found. C1 and D corals of *A. millepora* are both relatively thermo-tolerant (with D corals slightly more thermo-tolerant), and *Symbiodinium* A is a poor symbiotic partner for *A. millepora* with opportunistic characteristics. Trade-offs between thermo-tolerance and growth/survival rate vary between *A. millepora*-*Symbiodinium* associations, and differing environmental conditions can weaken or strengthen these trade-offs. The results of this study support the notion that symbiont shuffling [7], [21] is likely to play a major role in the response of this species to global warming. However, care has to be taken not to overestimate the potential of this response, as it is more likely that symbiont shuffling would only buy time rather than save this coral species from the impacts of climate change (see also [26], [27], [59]). The main question to be addressed now is how representative these findings are for other corals species, including other species within the genus *Acropora*. The availability of similarly detailed fitness information for many coral species will greatly enhance our ability to predict how corals will react to the increased heat-stress they face as a result of global warming.

## Materials and Methods

### Research locations

Two inshore reef locations were selected ca. 750 km apart: Magnetic Island (19.1 S, 147.5 E) in the central Great Barrier Reef (GBR), and Miall Island (23.1 S, 150.5 E) within the Keppel Islands group in the southern GBR. Note that throughout the text, we will refer to Miall Island as the Keppel Islands. The sites differ significantly in several aspects (Table 2). Furthermore, spawning times of *Acropora millepora* colonies differ by one month between the two locations, which made it possible to perform the experiments on both populations within a single year. The study was part of the research plan of the Australian Institute of Marine Science (Townsville, Australia) and the Great Barrier Reef Marine Park Authority (Townsville, Australia) supplied the necessary permits to collect and outplant the corals.

**Table 2:** Comparison of Magnetic Island and the Keppel Island field locations and their *A. millepora* populations.

Factor	Magnetic Island	Keppels Islands
Mean Summer Seawater Temperature	29.2±0.45^1^	27.0±0.50^1^
Bleaching threshold	31.2°C–5 days exposure	29.5°C–5 days exposure
	30.4°C–20 days exposure^2^	28.8–20 days exposure 2
Symbiont clade/type	D^1,3^	C2 (95%)+D (5%)^1,3^
Spawning time	October^4^	November^4^

1 [12].

2 [65].

3 [27].

4 This study.

### Coral host populations and *Symbiodinium* types

*Acropora millepora* was chosen because its relationship with *Symbiodinium* types at the two research locations was already established [12], [41]), the populations at Magnetic Island and the Keppel Islands are known to be genetically distinct (Smith-Keune & van Oppen 2006), and experience in raising specific coral-*Symbiodinium* associations was already available for this species [13].

*Symbiodinium* types were harvested from three coral species and three locations on the GBR (Details are provided as supporting information, Table S1). They were identified based on the nuclear ribosomal DNA internal transcribed spacer 1 (ITS1) region using a combination of Single Strand Conformation Polymorphism (SSCP) and DNA sequencing [44], [60]. Initially, five *Symbiodinium* types were selected for the inoculations of juvenile corals (designated as C1, C2*, C2, C• and D), as these are abundant on the GBR [12], [41], [44] and are therefore ecologically relevant. C1, C2, C2* and D are normally found in adult *A. millepora*; C1 and D are predominantly found at inshore, more turbid locations and C2/C2* more at cooler, clearer locations. So far, C• has not been found in *A. millepora* on the GBR or elsewhere, and is mostly found in maternally transmitting corals such as *Montipora* and *Porites* ssp. SSCP analyses revealed that the two *A. millepora* colonies from Davies reef, collected for their C2* type, harbored ∼50% *Symbiodinium* type C2* and ∼50% *Symbiodinium* type A (supporting information, Fig. S1). Clade A is very rare on the GBR [43], [44], and is mostly found in the southern GBR and higher latitude reefs [54]. The latter combination brought the total number of *Symbiodinium* types used in the inoculations to six; Four types were offered in isolation while C2* and A were administered as a 50–50 mixture.

All ITS1 sequences obtained were identical to sequences available in GenBank (A-AB207206, C1-AF380551, C2-AY643495, C2*-AY643497, C•-AY237300 and D-EU024793). ITS1 genotypes A, C1, C2, and C• correspond to ITS2 genotypes A1, C1, C3, and C15, respectively [27], [43], [45]. At several stages during the following 8 months of grow-out on the reef, a subset of the juvenile corals was genotyped from each group to verify that the symbiont type matched what had been experimentally offered.

### Preparation, outplanting and monitoring of juvenile corals

Juvenile corals were raised and outplanted following Cantin *et al.* [61]. Further details are given in the Supporting Materials and Methods S1. Juvenile corals raised from coral colonies originating from Magnetic Island were outplanted to Magnetic Island but not to the Keppel Islands due to logistical limitations. In contrast, juvenile corals raised from colonies originating from the Keppel Islands were outplanted to both Magnetic Island and the Keppel Islands.

The nomenclature of experimental groups consists of a three-letter code designating the location of the outplant, the location of the parental population, and the *Symbiodinium* type. For example, MKC1 means that the group was outplanted to Magnetic Island, and consisted of juveniles originating from the Keppel Islands population and *Symbiodinium* type C1.

The field locations were visited three times during the grow-out phase, which ran for 31 (Keppel Islands hosts) or 35 (Magnetic Island hosts) weeks. Details of the growth and survival measurements are given in the Supporting Materials and Methods S1. Briefly, growth was estimated from changes in two-dimensional surface area (averaged per tile-side) measured from scaled digital photos, taking care to use only single, non-fused colonies. Survival was determined by changes in colony number per tile-side over the experimental period.

### Laboratory heat-stress experiments

The design of the heat-stress experiments followed Berkelmans and van Oppen [12]. Tiles were divided over four temperature treatments with three replicate tanks (total of 12 tanks), with underwater light intensity of 120–150 µmol photons.m^−2^.s^−1^ provided by 400 W metal halide lamps (BLV, Germany). Each experimental group was represented by one tile per tank, and the number of coral juveniles on each tile ranged from 10 to 70 (average of 25). Further details are given in the Supporting Materials and Methods S1. Due to the limited light field, the experimental set-up could accommodate a maximum of four groups. In order to assess the thermo-tolerance of the maximum number of coral groups, two successive experiments were performed.

#### Experiment 1

Performed in May-June 2006, this experiment involved the coral groups MMC1, MKC1, MMD and MKD. Juvenile corals were acclimated at 27 °C (the ambient temperature at Magnetic Island in autumn) for 10 days with increasing photoperiod from 5L∶19D to 8L∶16D. The temperature was raised to the target temperatures over a period of three days (27° (control), 30.5°, 31.5° and 32.5°) and maintained for 18 days. During the experiment, the photo-period was further increased in two steps (Day 8 and 16) to 10L∶14D.

#### Experiment 2

Four additional coral groups, KKA, KKC1, KKD and MKC1, were heat-stressed in July 2006. Only three temperature treatments were performed because an insufficient number of colonies was available for four treatments. Juvenile corals were acclimated at 22°C (the approximate ambient temperature at the Keppel Islands in early spring) for 9 days with an increasing photo-period starting at 3L∶21D to 10L∶14D. At the end of the acclimation period, the temperature was raised to the target temperatures over a period of seven days (27° (control), 31° and 32.5°) and maintained for 15 days. Due to technical issues constraining the maximum temperature difference between treatments, the control group temperature was increased to 27°C.

### Photosynthetic performance

Photosynthetic performance (as an indicator of thermal stress) was assessed using a MAXI-imaging PAM (MAXI-iPAM; Walz, Germany). Details are given in the Supporting Materials and Methods S1. Briefly, the maximum and effective quantum yields (Fv/Fm and F/Fm', respectively) were measured, and the excitation pressure over photosystem II (Q) was calculated according to the formula described by Iglesias-Prieto *et al.* [39].

Q is a highly informative measure for photosynthetic performance that takes into account both the photochemical and non-photochemical processes [39]. It provides an indication of the ratio between open and closed reaction centres of photosystem II under the experimental irradiance level: a value of close to zero indicates that most of the reaction centres are open, suggesting light-limitation; a value close to one indicates that almost all reaction centres are closed, suggesting photo-inhibition. Although still poorly understood, thermal bleaching of corals is inherently associated with an accumulation of excitation pressure within PSII [62], [63]. Therefore, increases in Q over time to unusually high values, under constant light levels and accumulating heat-stress, are indicative of chronic photoinhibition and, therefore, a bleaching response [34].

### Real-time PCR and visual assessment

Six juvenile colonies were taken per experimental group/treatment (2 per tank) before heating started (experiment 2 only) and one day after the last PAM measurements, to determine relative *Symbiodinium* cell densities. For this, the real-time PCR assay based on actin genes and described in Mieog *et al.* [64] was followed, using SDS-based DNA extraction and normalization to coral surface area. New real-time PCR primers for *Symbiodinium* type A were developed following the method described in Mieog *et al.* [64]. More information about the real-time PCR assay is given as supporting information (Supporting Materials and Methods S1, Table S2 and Fig. S2). Densities were expressed in relative rather than absolute numbers, avoiding the estimation of DNA extraction efficiencies and actin gene copy numbers. This method assumes that extraction efficiencies were equal for all samples. *Symbiodinium* densities of the pre-stress (only available in the second heat-stress experiment) or control treatments were set to 100%.

Mortality unrelated to bleaching, which may have been caused by accidental abrasion of the coral juveniles during the cleaning of the tiles, was judged by the presence of patchy tissue necrosis. These individuals were immediately removed to avoid the spreading of any disease and were not included in the data. All colonies were visually scored at the end of the experiment as healthy, pale, or bleached. The red color of the terracotta tiles was used as a color reference, and the data was conservatively analyzed with an emphasis on bleached vs healthy/pale.

### Statistical analyses

For growth, mean colony surface areas were compared between coral groups. Colony surface areas were averaged per tile side to facilitate analyses and to be conservative. In the first test, all groups (except KKA) were compared at T = 31 weeks. KKA was left out of this analysis because no other A group was present to test for host population or environmental effects. The data for MMC1 and MMD were interpolated per tile side to T = 31 weeks by curve-fitting the data using all time points. The T = 31 data were log-transformed to correct for heteroscedacity of variances. A general linear model ANOVA was used, specifying the following fixed terms: Symbiont type, Host population, Outplant location, Symbiont type*Outplant location, Symbiont type*Host population.

To further analyze the effect of the three *Symbiodinium* types on growth at the Keppel Islands, a repeated measure model ANOVA was run on all data points (T = 6, 13 and 31 weeks). Data were averaged per tile side and log-transformed as before. Symbiont type was specified as the (fixed) predictor, with Time as the Within-Subjects factor. When a significant Symbiont type effect was found, a Fisher *post hoc* test was performed to determine which symbiont types were different.

Survival was analyzed for each outplant location with Kaplan-Meyer log-rank tests. As no satisfactory method of interpolation could be established for the survival data, pairwise comparisons of host populations were used for the groups outplanted to Magnetic Island (MMC1 x MMD and MKC1 x MKD) to test for an effect of Symbiont type. For the Keppel Islands, all groups were included in a first test for Symbiont type. Upon finding a significant effect, pairwise comparisons were performed to establish where the differences were located.

Analyses of the laboratory heat-stress experiments utilized PAM-fluorometry data and symbiont density data. Separate analyses were performed for each experiment since the stress-levels differed. Q fluorescence data was arcsine transformed and analyzed using repeated measures model ANOVAs. To correct for differences in colony number per tile, average Q values per tile were calculated. For experiment 1, a full factorial approach was used with Time as the Within-Subjects factor. The following fixed terms were specified: Temperature, Symbiont type, Host population. For experiment 2 the data were analyzed in two steps: first, KKC1 and MKC1 were analyzed, with Time as the Within-Subjects factor and Outplant location as the (fixed) predictor. If no significant differences were found, all groups were included in a second analysis with Time as the Within-Subjects factor and Temperature and Symbiont type specified as (fixed) predictors.

*Symbiodinium* density data were square-root transformed and analyzed using factorial ANOVAs. The same approach was used as described for the fluorescence data set.

## Supporting Information

Table S1 (0.03 MB DOC)

Table S2 (0.03 MB DOC)

Table S3 (0.05 MB DOC)

Supporting Materials and Methods S1 (0.03 MB DOC)

Figure S1 SSCP profiles of the six *Symbiodinium* types used for tank inoculations. Top line = *Symbiodinium* type, M = Marker of reference ITS1 sequences. C2* was found to be a mix of types C2* and A.
(0.08 MB DOC)

Figure S2 Overview of the partial *Symbiodinium* actin genes used in real-time PCR analyses. Top line gives position in bp from start of the alignment, left bar indicates *Symbiodinium* clade (O = overview of exons (E) and introns (I)). ï¿½ = present, − = absent. Arrows show the annealing sites of the actin primers given in Table S2.
(0.03 MB DOC)

Figure S3 PAM-results of heat-stress experiment 1. Effect of four different temperature regimes on the maximum quantum yield of four groups of juvenile corals. Juvenile corals harboring *Symbiodinium* C1 respond more strongly to the highest temperature than those harboring D. L:D = light-dark regime, # = target temperature is reached.
(0.06 MB DOC)

Figure S4 PAM-results of heat-stress experiment 2. Effect of three different temperature regimes on the maximum quantum yield of four groups of juvenile corals. Corals harboring *Symbiodinium* A respond more strongly to the highest temperature regime than those harboring either C1 or D. # = target temperature is reached.
(0.04 MB DOC)
