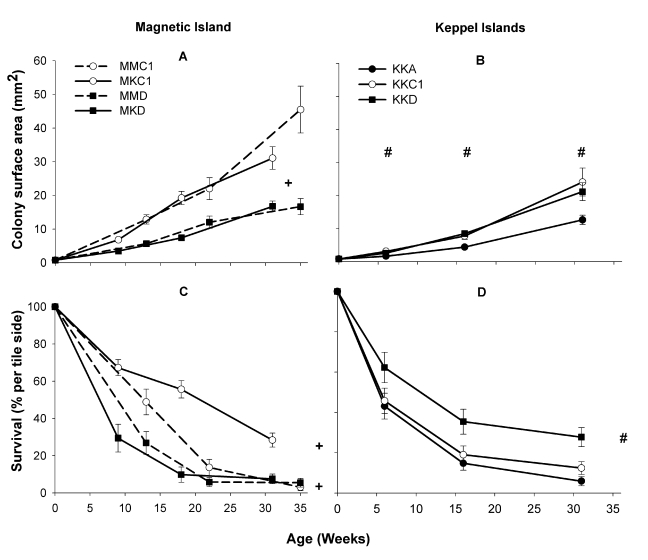# Correction: The Roles and Interactions of Symbiont, Host and Environment in Defining Coral Fitness

**DOI:** 10.1371/annotation/e06b31ef-6b29-44ae-aec1-1740daa93f4b

**Published:** 2009-08-13

**Authors:** Jos C. Mieog, Jeanine L. Olsen, Ray Berkelmans, Silvia A. Bleuler-Martinez, Bette L. Willis, Madeleine J. H. van Oppen

Figure 1 contains errors. Please see the corrected figure here: 

**Figure:**