# Supplementary material for: The Roles and Interactions of Symbiont, Host and Environment in Defining Coral Fitness
Source: PLoS One. 2009 Jul 24;4(7):e6364. doi: 10.1371/journal.pone.0006364 (PMC2710517; doi:10.1371/journal.pone.0006364)
Supplement: Table S1 — (0.03 MB DOC) [file pone.0006364.s001.doc]

**Table S1.** *Symbiodinium* inoculations***.*** The *Symbiodinium* type, species and origin (including position) of the coral colonies used as the source for the *Symbiodinium* inoculations.

| **Clade/Type*** | **Host species** | **Origin** | **Lat-Long** |
| --- | --- | --- | --- |
| C1 | *Acropora tenuis* | Magnetic Island | 19.1 S, 147.5 E |
| C2 | *Acropora millepora* | Keppel Islands | 23.1 S, 150.5 E |
| C2*/A | *Acropora millepora* | Davies Reef | 18.5 S, 147.1 E |
| C• | *Porites australiensis* | Magnetic Island | 19.1 S, 147.5 E |
| D | *Acropora millepora* | Magnetic Island | 19.1 S, 147.5 E |

**sensu* [12,66]
