# Supplementary material for: The Roles and Interactions of Symbiont, Host and Environment in Defining Coral Fitness
Source: PLoS One. 2009 Jul 24;4(7):e6364. doi: 10.1371/journal.pone.0006364 (PMC2710517; doi:10.1371/journal.pone.0006364)
Supplement: Table S2 — (0.03 MB DOC) [file pone.0006364.s002.doc]

**Table S2.** Primers used for *Symbiodinium* type A actin gene sequencing (a+b) and for *Symbiodinium* A, C and D real-time PCR analyses (c-h). Annealing sites of the primers are given in Fig. S2.

| **Primer name** | **Sequence (5’→3’)** |
| --- | --- |
| Primers for actin gene sequencing |  |
| a) Universal actin forward primer 1 | GGCTACTCCTTCACCACCAC |
| b) Clade A actin reverse primer | GCAGTCAGCTCCTTGGTCAT |
| Primers for *Symbiodinium* real-time PCR |  |
| c) A actin forward primer | GCCGGATGGCAACATCAT |
| d) A actin reverse primer | CCGGTTGTGGCAGGACAT |
| e) C actin forward primer^a^ | CAGGATGACACATGCTGATGAA |
| f) C actin reverse primer^a^ | AATTGATGGATTTGTTGGAACTTGT |
| g) D actin forward primer^a^ | GTGAAATTGCGCGTGACATC |
| h) D actin reverse primer^a^ | AGTGCTCCCACTGTCCAACC |

^a^ [64]
