# Supplementary material for: The Roles and Interactions of Symbiont, Host and Environment in Defining Coral Fitness
Source: PLoS One. 2009 Jul 24;4(7):e6364. doi: 10.1371/journal.pone.0006364 (PMC2710517; doi:10.1371/journal.pone.0006364)
Supplement: Table S3 — (0.05 MB DOC) [file pone.0006364.s003.doc]

**Table S3.** Temporal confirmation (by SSCP and sequencing) of *Symbiodinium* genotype in outplanted juvenile corals. See Materials and Methods section for group nomenclature. Juvenile corals that were inoculated with C2 and C• but failed to take them up are marked “un”. *Symbiodinium* types taken up in the field are marked in bold.

| **Age (Weeks)** | **Group** | **Nr. genotyped** | ***Symbiodinium* type** |
| --- | --- | --- | --- |
| **At Magnetic Island** | | | |
| 9 | MKC1 | 7 | All C1 |
| 9 | MKD | 12 | All D |
| 9 | MKA | 7 | All D |
| 9 | MKun | 13 | All **D** |
|  |  |  |  |
| 13 | MMC1 | 7 | All C1 |
| 13 | MMD | 12 | All D |
| 13 | MMA | 9 | All **D** |
| 13 | MMun | 19 | All **D** |
|  |  |  |  |
| 31 | MKC1 | 5 | All C1 |
| 31 | MKD | 8 | All D |
|  |  |  |  |
| 35 | MMC1 | 8 | All C1 |
| 35 | MMD | 8 | All D |
|  |  |  |  |
| **At Keppel Islands** | | | |
| 6 | KKA | 7 | All A |
| 6 | KKC1 | 7 | All C1 |
| 6 | KKD | 10 | All D |
| 6 | KKun | 6 | All **D** |
|  |  |  |  |
| 10 | KKA | 4 | 3 A, 1 **D** |
| 10 | KKC1 | 5 | All C1 |
|  |  |  |  |
| 13 | KKA | 8 | 5 A, 1 A+**D,** 1 A+**C1+D,** 1 **C1+D** |
| 13 | KKun | 12 | **11D, 1 C1+D** |
|  |  |  |  |
| 31 | KKA | 14 | 10 A, 3 A**+D,** 1 **C1+D** |
| 31 | KKC1 | 11 | All C1 |
| 31 | KKD | 11 | All D |
