# Supplementary material for: The Roles and Interactions of Symbiont, Host and Environment in Defining Coral Fitness
Source: PLoS One. 2009 Jul 24;4(7):e6364. doi: 10.1371/journal.pone.0006364 (PMC2710517; doi:10.1371/journal.pone.0006364)
Supplement: Supporting Materials and Methods S1 — (0.03 MB DOC) [file pone.0006364.s004.doc]

**SUPPORTING MATERIAL AND METHODS S1**

**Spawning of the two *A. millepora* populations**

Twelve colonies of *A. millepora* were transported from Magnetic Island to the Australian Institute of Marine Science (AIMS) in mid-October 2005, and kept in outdoor tanks. On the night of 20 Oct 2005, four corals showed pre-spawning signs (“setting” *sensu* Babcock *et al.* [67]) in the evening, and these were isolated in buckets. The released gametes were collected using 250 mL beakers and mixed together to ensure fertilization, before being transferred to 500 L larval rearing tanks.

This procedure was repeated a month later with *A. millepora* from the Keppel Islands. All twelve colonies spawned on the night of 16 November 2006. Gametes were collected from eight colonies for the experiment.

**Raising specific coral-*Symbiodinium* associations**

Embryos were reared in filtered seawater (1 μm) in five, round 500 L tanks. Densities were ca. 100,000 embryos/tank and water temperature was controlled at 27° C. After three days, (when the larvae were mobile), autoclaved terra-cotta tiles were placed on plastic racks to provide settlement surfaces. Tiles where either pre-conditioned at Magnetic Island for six weeks, or sprinkled with crushed coralline algae to induce settlement.

*Symbiodinium* inocula were prepared from the corals (Table S1) by airbrushing followed by centrifugation of the coral-algal slurry (350 g for 5 min). These were added to the tanks of azoothanthellate, juvenile larvae on the third and seventh day after spawning. Ca. 50 million freshly isolated algal cells were added to the ca. 500L of filtered seawater in the larval rearing tank during each inoculation. Because no *Symbiodinium* cells could be detected in the newly settled juveniles of tanks inoculated with C2*, C2 and C• after 12 days (using 6 squash preps per group, see below), an extra inoculation was performed for these groups.

At seventeen days after spawning, twenty squash preparations of coral polyps were made per tank and analyzed under a fluorescent microscope to check that *Symbiodinium* cells were present in the coral tissues. Sixteen squash preparations/group were stored in 100% ethanol for later genetic analysis.

**Outplanting**

Each tiles tile was labeled, photographed and mounted on a stainless steel rod that passed through a central hole in the tile. Up to nine tiles could be mounted per rod, plus two “edge”-tiles without coral juveniles. Tiles were separated by 2 cm spacers.

The tile-laden rods were transported to Magnetic Island and the Keppel Islands in 100 L plastic containers filled with seawater; transport to the Keppel Islands included a continuous flow-through system. Rods were fixed horizontally between two star-pickets and suspended ~40 cm above the substratum in a zone where *Acropora spp.* were present.

**Growth and survival measurements**

Tile-laden rods were collected around noon and transported to the shore (close to the field site) where they were kept in plastic 100 L containers containing ~30 L of seawater (two rods/container). A small pump was added to provide water circulation. Tiles were removed from the rods one by one, placed in a plastic bowl containing seawater and photographed from a fixed distance. Tiles were returned to the field the following morning.

Growth was estimated from changes in two-dimensional surface area measured from the digital photos using the software package Image-J (NIH Image). Care was taken to use only single, non-fused colonies. The total number of tile-sides measured ranged from 8 to 15; the number of colonies per time point ranged from 34 to 342.

Survival was determined by changes in colony number per tile side over the experimental period. Up to 42 single polyps (numbers ranged from 5 to 42 polyps) were randomly selected from 10 to 14 tile sides on pre-field pictures (total numbers ranged from 165 to 247 colonies/group), and their survival or mortality was noted over the different time-points.

**Heat-stress experiments**

Tiles were shipped from the field to AIMS wrapped in wet bubble wrap and packed in insulated containers (no seawater added). Once at the institute, all growth other than the coral colonies was removed from the tiles using forceps and scalpels. Tiles were placed on plastic racks in 50 L tanks, and cleaned on an average of every four days.

The tanks were supplied with fresh filtered (1 μm) seawater via a flow-through system at a rate of 1 L.min^-1^. Two small pumps per tank provided water movement. Water temperature was computer-controlled to within 0.05 °C [68]. Light was provided by one metal halide lamp per tank (10,000 K color temperature, BLV Germany) with a spectrum suitable for photosynthesis, at an underwater light intensity of 120-150 μmol photons.m^-2^.s^-1^. Compared to the maximum natural light levels for adult corals at the two field sites, these light levels are low (Magnetic Island: 300-700 μmol photons.m^-2^.s^-1^ at midday; Keppels: 150-800μmol photons.m^-2^.s^-1^ at midday (Berkelmans unpub. data)). However, the coral juveniles were most likely acclimated to lower light levels than adult corals because of their cryptic nature: they grew on the vertical-positioned tile sides, and these tile sides were overgrown with turf and other algae, effectively shading the coral juveniles. In addition, in the field corals experience the full range of diurnal variation with high light levels experienced for only a few hours around midday, whereas the light levels in the heat-stress experiments were constant.

**PAM-fluorometry**

The MAXI-iPAM (Walz, Germany) allowed a whole tile-side to be measured at once. Tiles were kept reasonably clean to avoid interference from turf algae. ImagingWin software was used for all measurements. For dark-adapted measurements of maximum quantum yield (Fv/Fm), tiles were kept for at least 14 hours in darkness before being moved from the tanks to the MAXI-iPAM under fluorescent light conditions of <2 μmol photons.m^-2^.s^-1^. Light-adapted measurements of effective quantum yield (F/Fm’) were taken 2-4 hours after the photoperiod had started. Tiles were placed (one at a time) under the MAXI-iPAM and exposed to 1 min actinic light of 146 μmol photons.m^-2^.s^-1^ before measuring F/Fm’. During post-measurement analyses, an Area-Of-Interest (standard-sized area in the center of the colony) was assigned to each coral colony for each whole-tile measurement, to obtain Fv/Fm and F/Fm’ per colony.

**Real-time PCR measurements**

The actin gene of *Symbiodinium* type A was sequenced and real-time PCR primers were designed and tested as described for the *Symbiodinium* C and D actin real-time PCR primers in Mieog *et al.* [64]. Primers for sequencing and real-time PCR are given in Table S2. The real-time PCR primers were found to be clade A specific, and amplified with near 100% efficiency. The position of the A-specific real-time PCR primers in relation to the C and D-specific primers is given in Fig. S2.
