# Supplementary material for: The Roles and Interactions of Symbiont, Host and Environment in Defining Coral Fitness
Source: PLoS One. 2009 Jul 24;4(7):e6364. doi: 10.1371/journal.pone.0006364 (PMC2710517; doi:10.1371/journal.pone.0006364)
Supplement: Figure S4 — PAM-results of heat-stress experiment 2. Effect of three different temperature regimes on the maximum quantum yield of four groups of juvenile corals. Corals harboring Symbiodinium A respond more strongly to the highest temperature regime than those harboring either C1 or D. # = target temperature is reached. (0.04 MB DOC) [file pone.0006364.s008.doc]

a) 27°C

b) 31°C

c) 32.5°C
