# Supplementary material for: The Roles and Interactions of Symbiont, Host and Environment in Defining Coral Fitness
Source: PLoS One. 2009 Jul 24;4(7):e6364. doi: 10.1371/journal.pone.0006364 (PMC2710517; doi:10.1371/journal.pone.0006364)
Supplement: Supplementary file 7 [file pone.0006364.s006.doc]

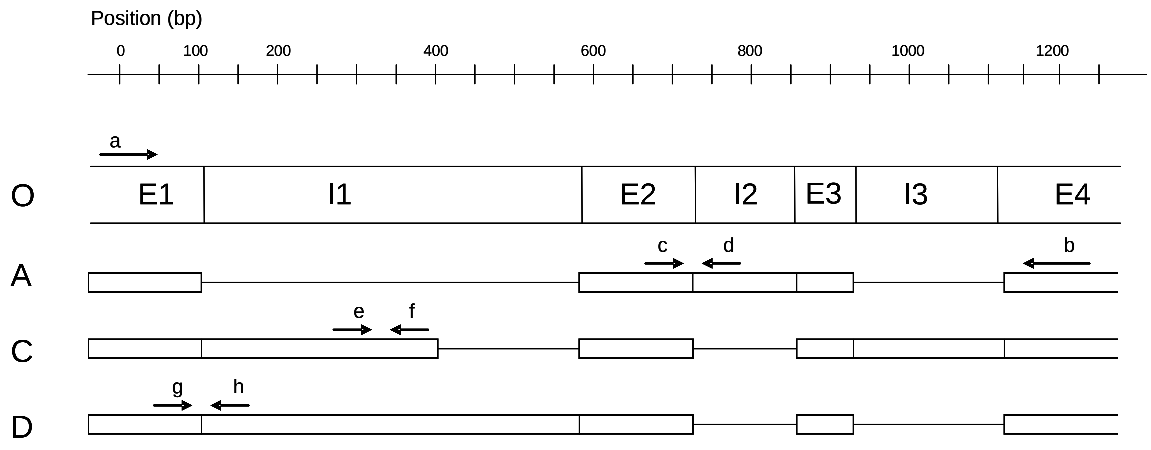
Position (bp)
0
100
200
400
600
800
1000
1200
a
O
E1
I1
E2
I2
E3
I3
E4
A
c
d
b
C
e
f
D
g
h
