# Supplementary material for: The Roles and Interactions of Symbiont, Host and Environment in Defining Coral Fitness
Source: PLoS One. 2009 Jul 24;4(7):e6364. doi: 10.1371/journal.pone.0006364 (PMC2710517; doi:10.1371/journal.pone.0006364)
Supplement: Supplementary file 8 [file pone.0006364.s007.doc]

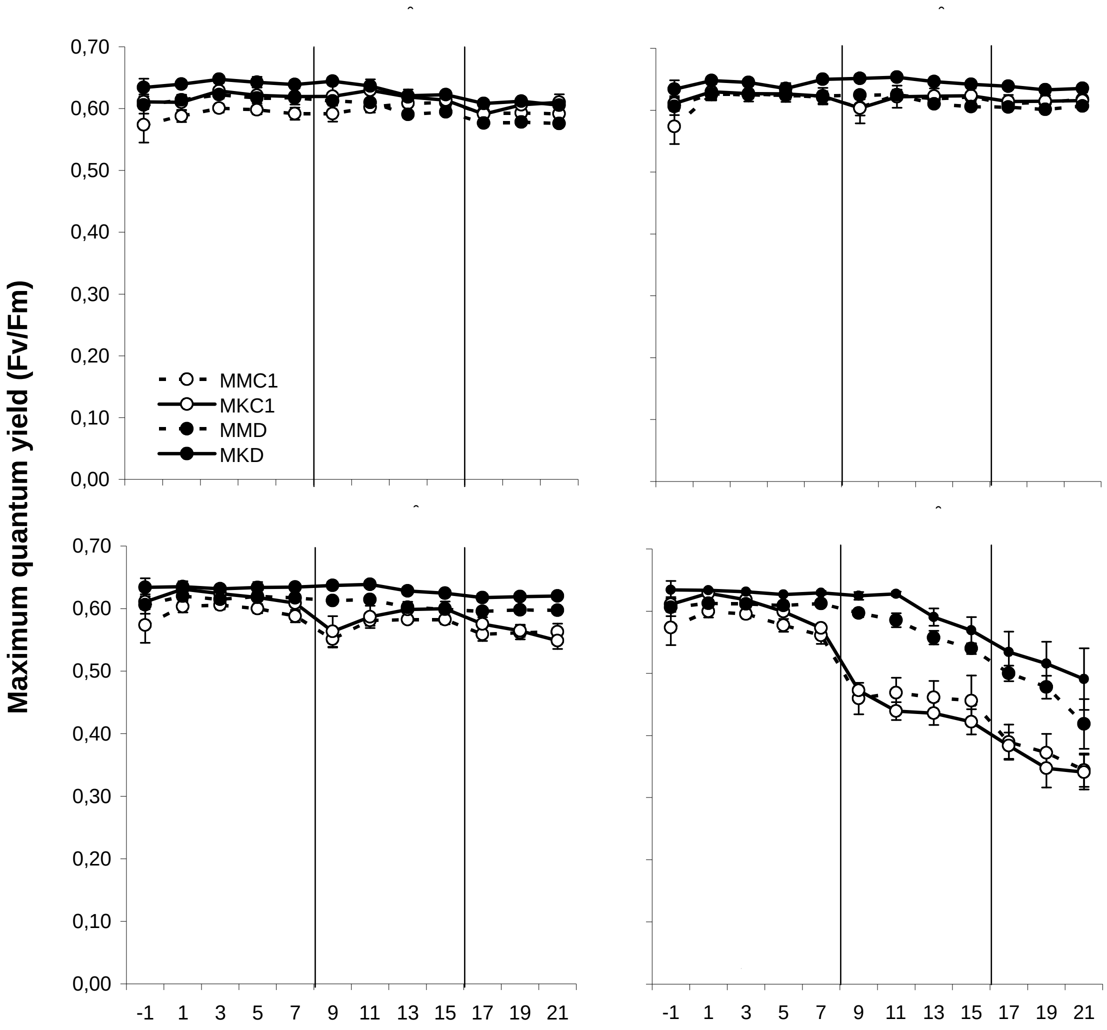

Maximum quantum yield (Fv/Fm)
0,70
0,60
0,50
0,40
0,30
0,20
0,10
0,00
MMC1
MKC1
MMD
MKD
-1 1 3 5 7 9 11 13 15 17 19 21
